# Adipocyte inflammation and pathogenesis of viral pneumonias: an overlooked contribution

**DOI:** 10.1038/s41385-021-00404-8

**Published:** 2021-05-06

**Authors:** Pablo C. Alarcon, Michelle S.M.A. Damen, Rajat Madan, George S. Deepe, Paul Spearman, Sing Sing Way, Senad Divanovic

**Affiliations:** 1 Department of Pediatrics, University of Cincinnati College of Medicine, Cincinnati, OH USA; 2 Divisions of Immunobiology, Cincinnati Children’s Hospital Medical Center, Cincinnati, OH USA; 3 Medical Scientist Training Program, Cincinnati, OH USA; 4 Immunology Graduate Program Cincinnati Children’s Hospital Medical Center and the University of Cincinnati College of Medicine, Cincinnati, OH USA; 5 Division of Infectious Diseases, Department of Internal Medicine, University of Cincinnati College of Medicine, Cincinnati, OH USA; 6 Veterans Affairs Medical Center, Cincinnati, OH USA; 7 Divisions of Infectious Diseases, Cincinnati Children’s Hospital Medical Center, Cincinnati, OH USA; 8 Center for Inflammation and Tolerance, Cincinnati Children’s Hospital Medical Center, Cincinnati, OH USA

## Abstract

Epidemiological evidence establishes obesity as an independent risk factor for increased susceptibility and severity to viral respiratory pneumonias associated with H1N1 influenza and SARS-CoV-2 pandemics. Given the global obesity prevalence, a better understanding of the mechanisms behind obese susceptibility to infection is imperative. Altered immune cell metabolism and function are often perceived as a key causative factor of dysregulated inflammation. However, the contribution of adipocytes, the dominantly altered cell type in obesity with broad inflammatory properties, to infectious disease pathogenesis remains largely ignored. Thus, skewing of adipocyte-intrinsic cellular metabolism may lead to the development of pathogenic inflammatory adipocytes, which shape the overall immune responses by contributing to either premature immunosenescence, delayed hyperinflammation, or cytokine storm in infections. In this review, we discuss the underappreciated contribution of adipocyte cellular metabolism and adipocyte-produced mediators on immune system modulation and how such interplay may modify disease susceptibility and pathogenesis of influenza and SARS-CoV-2 infections in obese individuals.

## Introduction

The obesity pandemic continues unabated, afflicting >500 million people worldwide. The U.S. has the highest mean adult body mass index (BMI) among high-income countries,^1^ with 42.4% of adults considered obese (BMI 30-35).^2^ It is anticipated that roughly half of the U.S. population will be obese by 2030, with morbid obesity (BMI > 35) impacting an estimated 24% of Americans.^3^ Obesity promotes chronic low-grade systemic and tissue inflammation that is central to the pathogenesis of various metabolic diseases including type 2 diabetes (T2D), non-alcoholic fatty liver disease (NAFLD), cardiovascular disease (CVD), and various cancers.^4^ Although commonly overlooked, obesity is also linked with elevated susceptibility and risk of developing serious complications to viral pneumonias.^5^ Epidemiological evidence from the 2009 H1N1 influenza pandemic established obesity as an independent risk factor for increased disease severity and mortality,^6^ including pulmonary and cardiovascular damage.^7^ Similar clinical observations in obese individuals are reported in SARS-CoV-2 (colloquially known as COVID-19) pandemic. Despite the clinical significance, the key cellular and molecular mechanisms underlying obese susceptibility to infection are not well defined.

Immune cells are the conventional source of inflammatory milieu in obesity. Intuitively, immune cell contributions to obesity-associated susceptibility to infections is the most well-examined to date. Alterations in the systemic and tissue inflammatory milieu impact cellular metabolic pathways and inflammatory vigor.^8^ Obesity-associated dysregulation of immune responses, inflammatory vigor,^9^ and adipose tissue (AT) immune cell infiltration and function^10–12^ play major roles in infectious disease pathogenesis.^13,14^ However, this dogmatic view tends to overlook the contribution of the primary cell type in AT: the adipocyte. Adipocytes, akin to immune cells, can impact the immune system locally and systemically^15,16^ via underappreciated utility of multiple immune-modulatory pathways.^17,18^ In this context, adipocyte cellular metabolism and inflammatory phenotype are interconnected.^19^ Thus, the role of adipocyte cellular metabolism and inflammatory/pathologic capacity in instructing immune responsiveness to viral pathogens is of potential significance.

Here we briefly summarize the mounting clinical evidence supporting the premise that obesity is an independent risk factor for infectious disease morbidity and mortality, with emphasis on influenza and SARS-CoV-2. While the impact of obesity on immune cells in influenza has been comprehensively reviewed,^13^ such studies in SARS-CoV-2 are nascent. Importantly, the focus on adipocyte inflammatory contributions in the context of either infection is finite. Hence, by drawing parallels between influenza and SARS-CoV-2 outcomes in obesity, here we emphasize the need for additional studies to improve our understanding of how obesity shapes outcomes of viral pneumonias. Thus, in light of the dominant impact of obesity on adipocyte function, we discuss the understudied immune and inflammatory capacities of adipocytes, the methods in which they communicate with the immune system, and the known pathologies to which these responses contribute. We then highlight the contribution of adipocyte cellular metabolism to immune system dysfunctions. Lastly, we invoke the idea of “pathogenic adipocytes,” an adipocyte population with altered metabolic and inflammatory states which potentially arises in obesity and detrimentally impacts immune responses to infection.

## Epidemiological evidence

Clinical studies have recently begun to explore the role of obesity in infection. Although few studies have invoked a protective role,^20,21^ the majority of epidemiological data points toward the detrimental effects of obesity on infection susceptibility and disease severity.^5,14,22,23^ The variety of pertinent infections is broad, and ranges from pandemic-causing strains of pathogens to common community- and hospital-acquired infections (Table 1). For example, obese individuals have higher incidences of community-acquired skin, fungal foot, orofacial, acute respiratory (e.g., pertussis), gastrointestinal (e.g., *H. pylori*), and urinary tract infections (particularly in obese pregnant and post-partum women).^22,24,25^ Obese individuals also have an increased likelihood of contracting nosocomial infections, such as post-surgical site and catheter-acquired infections, which are attributed to metabolic factors such as hyperglycemia and diabetes.^22,26^ Further, while in critical care, obese individuals are at an elevated risk of developing and succumbing to life-threatening conditions (e.g., invasive candidiasis and *E. coli* bloodstream infections).^27,28^ However, while obese individuals have an increased risk of developing sepsis from infection,^29^ they are less likely to die from it.^30^ While most of these studies have been conducted in adults, congruent effects are observed in adolescents.^31^

**Table 1:** Infections with a clinically reported elevated risk in obesity.

Clinical category	Infection
Pandemic-associated	H1N1 influenza^6^; SARS^33^; MERS^34^; COVID-19 (SARS-CoV-2)^36–38^
Community-acquired	Seasonal influenza A^32^; Whooping cough (*Bordetella pertussis*)^24^; acute respiratory^25^; skin (*S. aureus*), fungal foot, orofacial, gastrointestinal (*H. pylori*), urinary tract^22^
Nosocomial	Aspiration pneumonia,^22^ post-surgical site (*S. aureus*),^26^ invasive candidiasis^27^; catheter-acquired, bloodstream (*E. coli*)^28^

Obesity’s impact on disease severity isn’t limited to common infections. While frequently linked to worsened disease severity during seasonal influenza A virus infections,^32^ obesity was also established as an independent risk factor for severe pulmonary disease and mortality during the 2009 H1N1 influenza pandemic.^6^ Subsequently, obesity was associated with elevated disease severity in coronavirus-induced outbreaks including Severe Acute Respiratory Syndrome (SARS)^33^ and Middle East Respiratory Syndrome (MERS).^34^ Most recently, systematic reviews of Coronavirus Disease 2019 (SARS-CoV-2; COVID-19) cases revealed that obese individuals are at an increased risk for hospitalization, respiratory failure, ICU admission, mechanical intubation, and mortality.^35–38^ Both H1N1 influenza and SARS-CoV-2 viral shedding is also prolonged in obese individuals,^32,39^ highlighting the negative impact obesity can have on efforts to control spread of pandemic strains of disease. Further, elevated risk of progression to severe pneumonia due to SARS-CoV-2 and influenza is coupled with obesity-associated metabolic sequelae, including T2D,^40,41^ CVD,^7,42^ and NAFLD.^43,44,45^ Given the overlaps between disease susceptibility and pathogenesis in either influenza and SARS-CoV-2 infection, inciting the discussion of potential obesity-impacted cellular and molecular mechanisms could promote the discovery and development of novel therapeutics.

## Adipocytes: altered by obesity

Traditionally, adipocytes play a key role in energy homeostasis.^46^ However, recent insights have broadened our knowledge of adipocytes as important endocrine and inflammatory cells^47^ with immune cell-like inflammatory properties.^18^ In fact, it is now well appreciated that adipocytes can take either beneficial or detrimental fates in disease.^17^ Adipocytes can sense and respond to bacterial, fungal, or viral components via expression of various TLRs,^48^ and adipocytes can present antigens to surrounding immune cells via MHC Class II expression.^49^ Adipocyte-derived secretory chemokines and acute-phase reactants, such as SAA3-α1 acid glycoprotein, CRP-relative pentraxin-3, and lipocalins can amplify local immune cell inflammatory cytokine expression.^50^ The role of adipocytes in systemic inflammation was emphasized during the “fatless” study, in which inactivation of adipocytes resulted in a strongly reduced septic LPS response.^51^ Adipocyte-secreted factors (e.g., adiponectin, leptin) impact systemic and tissue function and cellular metabolism resulting in altered immune cell responsiveness to external stimuli^52^ and AT inflammation.^17^ Adiponectin activity is viewed as anti-inflammatory, as it inhibits neutrophil ROS production, promotes macrophage IL-10 secretion, limits TLR-mediated NF-kB activation, and reduces Th1 T-cell polarization.^53,54^ Conversely, leptin is viewed as a pro-inflammatory mediator, as it enhances neutrophil ROS production and ICAM-1 expression, promotes macrophage proinflammatory cytokine (e.g., TNF, IL-6, IL-12) production, and skews T-cell polarization toward Th1 and Th17 subtypes.^55^

However, this adipocyte-immune cell interplay is modified by obesity. Systemically, obesity’s impact on various immune cells, summarized in Table 2, has been reviewed.^56^ In brief, obesity increases numbers and inflammatory capacity of circulating monocytes, macrophages, neutrophils, and Th1/Th17 CD4^+^ T cells.^57–59^ In contrast, the effector functions of eosinophils, Natural Killer (NK) cells, Dendritic Cells (DC), CD8^+^ T cells, and B cells are decreased.^60–65^ Within AT, obesity-associated adipocyte hypertrophy and hyperplasia leads to extensive AT remodeling that includes impaired AT angiogenesis, extracellular matrix protein deposition, and hypoxia-induced adipocyte pyroptosis.^66^ In response to pyroptotic adipocytes, recruited AT monocytes and macrophages increase AT inflammation.^67,68^ Further, direct communication between adipocytes and AT-resident T cells via MHC-II^49^ and macrophages via IL-1β^69^ instigate AT inflammation. This ultimately results in the surviving hypertrophic adipocytes having decreased adiponectin^70^ and increased leptin secretion.^71,72^ Obesity also impairs adiponectin exocytosis^73^ and decreases adiponectin receptor surface expression,^74^ while increased adipocyte leptin secretion is associated with changes in insulin regulation^75^ and IL-6 expression in AT.^76^ Despite such advances, further studies are needed to establish direct molecular mechanisms underlying adiponectin and leptin secretion in obesity. Adipocyte release of free fatty acids (FFA) is also enhanced in the obese state. Increased FFA levels promote inflammatory processes within monocytes and macrophages.^77^ In contrast, excessive exposure to FFA can decrease the cytotoxic capabilities of NK cells^78^ and cytotoxic T cells.^79^

**Table 2:** Effect of obesity on immune cell populations.

Immune cell	Within circulating populations	Within adipose tissue populations
Macrophage	• Increase total numbers of circulating monocytes and macrophages; increase proinflammatory populations^57^	• Increased infiltration and proliferation^185^ • Skewing toward proinflammatory M1 subtype, formation of “crown-like structures” within AT^84^
Neutrophil	• Elevated cytokine, reactive oxygen species, and neutrophil extracellular trap production^59^	• Early infiltration during obesity^186^ • Pro-inflammatory skewing^83^
Natural killer	• Decreased total numbers, reduced cytotoxic potential^61^ • Senescence in splenic populations^93^	• Increased infiltrating numbers and inflammatory capacity; polarize macrophages toward M1^187^
Dendritic Cell	• Impaired responsiveness to TLR agonists and airway allergens^62^ • Decreased ability to induce naïve T-cell proliferation^63^	• Increased infiltrating numbers, induce CD4^+^ Th17 differentiation^188^
B cell	• Impacted bone marrow precursor population^189^ • Decreased B cell antibody effector function and IL-10 production^64^ • Leptin-associated senescence^95^	• Increased proinflammatory B Cell recruitment^173^ • Exacerbate chronic inflammation^174^
CD8^+^ T cell	• Decreased total numbers^65^ • PD-1 expression mediated exhaustion, limited cytotoxic potential^14^ • Reduction of surface CD28, induction of senescence^94^	• Early infiltration during obesity, recruit macrophages to AT, contribute to AT inflammation^85^
CD4^+^ T cell	• Impacted total numbers^190^ • Increased Th1 and Th17 polarization^60^	• Favored proinflammatory Th1 and Th17 polarization^60^ • Th1 subset contributes to AT inflammation^86^ • Suppressed Th2 and Treg presence^191^

Recent reports also show that adipocytes secrete various proinflammatory mediators (e.g., IL-6, TNF, IL-1β) in response to external stimuli and that such responsiveness is further exacerbated in obesity.^19^ Locally, adipocyte IL-6 production regulates macrophage infiltration into AT.^80^ Systemically, IL-6 is elevated in the serum of obese individuals,^81^ with one-third of circulating IL-6 calculated to originate from AT.^82^ These interactions ultimately result in macrophages, neutrophils, and Th1/Th17 CD4^+^ T-cell skewing toward proinflammatory states and induction of AT inflammation.^69,83,84^ In contrast to their circulating counterparts, tissue NK, DC, CD8^+^ T, memory T, and B cells also gain proinflammatory phenotypes within AT.^83,85,86^ Interestingly, reduction of eosinophils within AT in obesity^87^ is linked with overall AT inflammation, as these cells regulate AT inflammation^88^ and promote the development of anti-inflammatory macrophages.^89^ Together, these findings invoke the importance of adipocyte contributions to an AT inflammatory microenvironment and immune cells function in obesity. How adipocyte-altered immune cell function within AT shapes the overall responses to an infection remains unanswered.

## Obesity and the immune system

Both adipocyte and immune cell inflammatory output contribute to increased systemic proinflammatory cytokine (e.g., IL-6, TNF, C-reactive protein) levels in obese individuals.^11,82^ However, the mechanisms connecting chronic low-grade inflammation and immune dysfunction in obesity remain undefined. Other disease states associated with chronic, low-grade inflammation such as aging^90^ and some cancers^91^ are linked with immune cell exhaustion and decline in immune cell function. This impaired immunological state robustly impacts host’s responses to pathogens and vaccine efficacies.^92^ Hence, obesity-associated chronic inflammation may similarly favor immune cell exhaustion/tolerance resulting in a premature immunosenescent-like state and dampened response to pathogens. Notably, obesity leads to splenic NK senescence, resembling changes seen in aging.^93^ Further, plasma from obese individuals is sufficient to induce senescence in cytotoxic T cells,^94^ and increased leptin levels (hallmark of obesity) induce B cell senescense.^95^ Altered immune responses of these cells to influenza in obesity^96^ may lead to impaired control of viral replication and increased disease severity. Whether obesity-induced senescence also impacts cellular regulatory process of inflammatory cascades^97,98^ in adipocytes should be examined, given their ability to directly respond to infection.^99^ If so, it is plausible that during influenza infection, immune cell senescence might contribute to increased viral load, whereupon dysregulated and continuous hyperinflammation by non-hematopoietic cells shapes inflammation-induced lung damage^13^ and impairs wound healing.^100^

In contrast, obesity-associated chronic immune activation could lead to an increased propensity of developing a dysregulated hyperinflammatory state that results in a “cytokine storm.” Cytokine storm is typically associated with severe or fatal immune reactions to pathogens or therapeutic interventions.^101,102^ Thus, obesity-mediated increases of inflammatory propensity in immune cells outlined in Table 2 might contribute to increased likelihood of developing cytokine storm. Obese mice which received stimulatory immunotherapy (anti-CD40/IL-2) develop lethal cytokine storm at an elevated rate.^103^ Similarly, obese mice, compared to lean counterparts, had exacerbated cytokine storm in response to *Francisella tularensis* infection.^104^ One potential mechanism lies within the ability of type I interferons (IFNs) to promote cytokine storm and viral-induced sepsis.^105^ Type I IFNs are independently induced by both obesity and viral infection.^106,107^ Further, type I IFN priming of immune cell^108–110^ or adipocyte^19^ amplifies their inflammatory vigor. Thus, additional studies focused on how obesity- and infection-elevated type I IFNs impact cellular inflammatory propensity may reveal novel molecular pathways by which obesity skews systemic inflammation toward a cytokine storm-susceptible state.

**Fig. 1:**
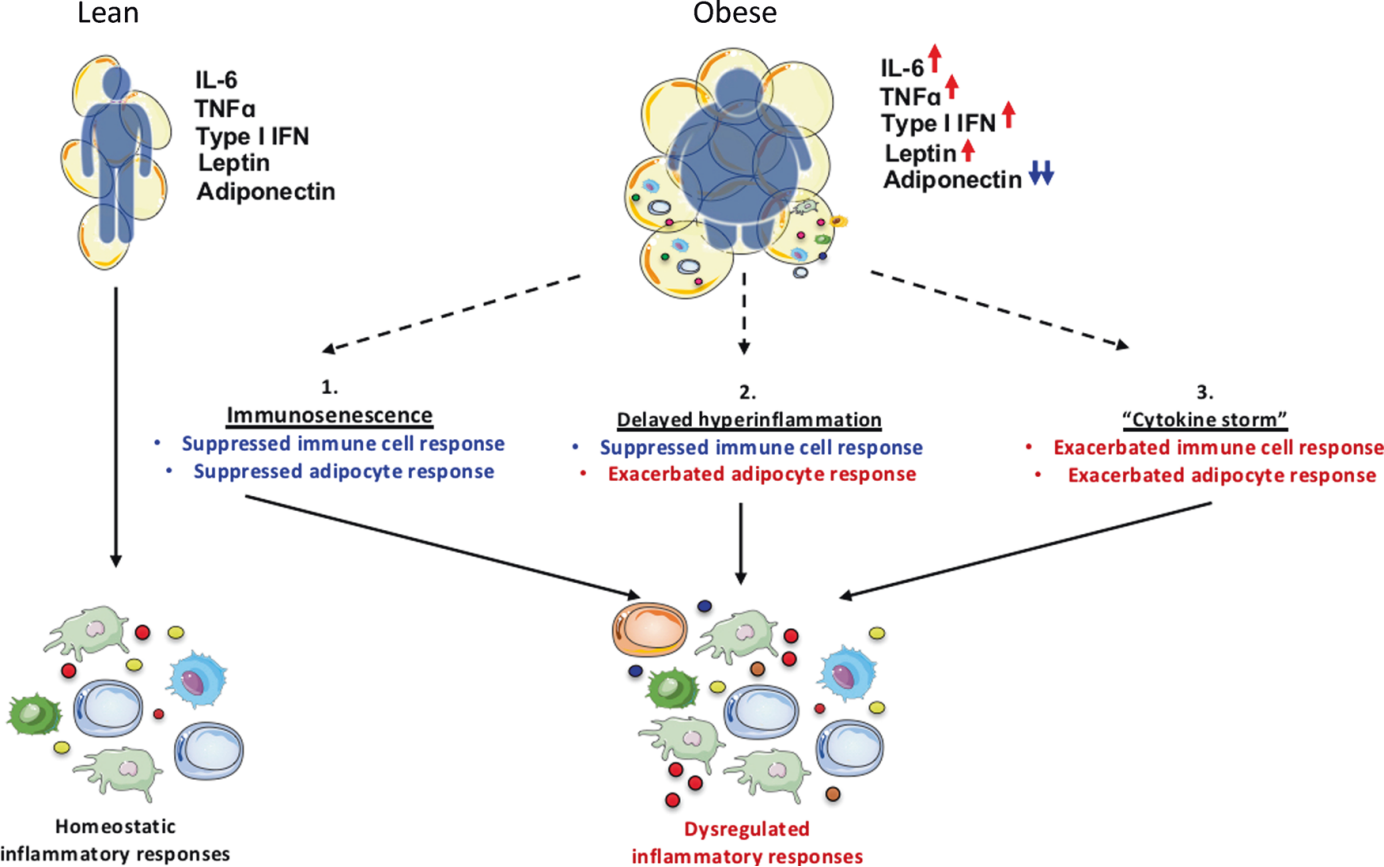
Obesity-altered adipocytes promote dysregulated inflammatory responses in infection. Adipocyte-secreted factors (e.g., Adiponectin, Leptin, Type I IFNs, and IL-6) contribute to homeostatic immune responses and appropriate immune defense mechanisms against infectious agents resulting in pathogen clearance in the lean state. Obesity-associated chronic inflammation leads to expansion in adipocyte number and size. These alterations in adipose tissue unlocks a proinflammatory skewing of adipocyte phenotype and leads to dysregulated secretion of adipocyte-produced mediators (increased in red, decreased in blue). Hence, obesity-dependent changes in adipocyte function can contribute to the immune system being in a state of: (1) Immunosenescence (suppressed immune response against pathogens); (2) Delayed immune inflammation (reduced pathogen clearance and compensatory exacerbated adipocyte inflammation); and (3) “Cytokine storm” (uncontrolled proinflammatory response and tissue damage). Overall, each altered inflammatory state in obesity could individually or synergistically shape immune responses, culminating in worsened disease pathology and increased morbidity and mortality.

While immunosenescence, delayed hyperinflammatory responses, and cytokine storm theories in obesity seem diametrically opposed, they are not necessarily mutually exclusive. The presence of a delayed immune response potentially bridges initial lack of responsiveness and an ultimately excessive response. As established earlier, not all immune cells are impacted in the same manner by obesity, and these effects may differ depending on organ or microenvironment where the immune cell resides. Increased susceptibility to infection in obese individuals may be dependent on the complex interplay between premature immunosenescence, delayed hyperinflammation by non-immune cells, and combined immune and non-immune cell-dependent cytokine storm. It is also important to consider the contribution of adipocyte inflammatory capacities on the immune system. Adipocytes respond to the elevated circulating endotoxemia present in obese individuals^111,112^ and communicate with immune cells.^113^ Thus, it is likely that preferential inflammatory skewing of adipocytes contributes to these aforementioned immunomodulatory effects of obesity via their secreted factors that in unison shape AT and systemic inflammation (Fig. 1). Whether these effects are pathogen-specific remains to be defined. These observations collectively invoke a fine nuance between obesity-associated chronic inflammation and immune cell inflammatory modulation—something that requires in-depth investigation and may be linked to adipocyte function in obesity and infection.

## Adipocytes and Infection: Pathogenic Links

The cellular and molecular mechanisms underlining the low-grade inflammatory state in obese individuals are not fully understood. However, alterations in cellular metabolism might provide a pathogenic link, as they are critical drivers of immune cell inflammatory function.^114–116^ Various studies have emphasized T cell and macrophage capabilities to sense environmental cues, quickly changing and adapting their cellular metabolic pathways to meet necessary energy demands and reshape their inflammatory/immune functions.^117–123^ Further, the contribution of AT macrophages and T cells pro‐inflammatory states to the pathogenesis of obesity-associated metabolic derangements is well established.^84,120,121,124^ Findings that in vivo alterations of multiple cellular metabolic pathways (e.g., glycolysis, TCA, OXPHOS) ameliorate inflammatory disease severity^120,125,126^ corroborate this postulated interplay between immune cell metabolism, inflammatory phenotype, and systemic inflammation. Thus, how obesity-associated metabolic alterations of immune cells and adipocytes influence infectious disease pathogenesis should be examined.

Sensing of Type I IFNs produced in response to viral infections shapes cellular function of T cells and macrophages via engagement of the ubiquitously expressed IFNAR receptors. Subsequent activation of the Janus kinase–signal transducer and activator of transcription (JAK–STAT) signaling pathway results in amplification of proinflammatory/antiviral gene expression.^127^ The ability of a type I IFNs to divergently impact immune cell function has been linked with altered disease pathology. For example, acute type I IFN production enhances DC maturation and monocyte recruitment and activation to sites of infection, which contributes to T-cell activation and establishment of anti-viral responses.^128^ In contrast, macrophages exposed to IFNβ show suppressed expression of the IFNγ receptor leading to lower IFNγ responsiveness and suppressed phagocytotic effects.^129^ A recent report suggests that type I IFN sensing by adipocytes resulted in approximately 30% gene expression profile conversion to that found in macrophages and was sufficient to alter adipocyte glycolysis and unlock adipocyte inflammatory potential,^19^ emphasizing the intimate relationship between these two processes. Additional recent reviews have also highlighted the relevance of immunometabolism in the function of other immune cells.^8,130^ Thus, the interplay between AT resident/infiltrating immune cells and adipocytes might constitute a key process in shaping cellular metabolism and inflammatory output of both cell types. Importantly, obesity-driven changes in metabolic pathways lead to altered inflammatory phenotypes in immune cells and adipocytes. These findings have led to an appreciation that distinct bioenergetics profiles not only guide immune cell pathogenic functions,^131–133^ but may similarly favor induction of a “pathogenic adipocyte” population—something that could be particularly deleterious to immune responses in an obesity-altered state. Whereas the roles of multiple metabolic pathways (e.g., glycolysis, OXPHOS, TCA cycle, pentose phosphate pathway, fatty acid oxidation/synthesis) in inflammation, cell proliferation, phenotype, and metabolic derangements have been examined in immune cells (Table 3), analysis of these pathways in adipocytes, and specifically obese-adipocytes, are highly limited (Table 4). Thus, given the availability of resources employed in interrogation of metabolic mechanisms underlying immune cell-driven disease pathogenesis, extension of their application to adipocytes is feasible and needed.

**Table 3:** Impact of metabolic pathways on immune cell inflammation.

Cell type	Metabolic pathway and impact
Monocytes	• Glycolysis: triggers increased phagocytic capacity^192^
Macrophages	M1 phenotype:
	• Glycolysis; supports proinflammatory phenotype (cytokines, chemokines) and rapid antibacterial response^193^
	• TCA/oxidative phosphorylation; supports phagocytic capacity, prolonged antihelminth response^194^
	• Fatty acid synthesis; supports proinflammatory phenotype^193^
	• Amino acid metabolism; supports proliferation and nitric oxide production^193–196^
	• Pentose Phosphate Pathway; essential for ROS/NO response and control of parasite replication^196^
	M2 phenotype:
	• Fatty acid oxidation; inhibiting inflammatory signals^193^
T cells	Effector T cell:
	• Glycolysis; needed for Th17 function (blocking glycolysis causes switch to T-regs and anti-inflammatory responses^194,197,198^
	• Fatty acid synthesis: support proliferation and regulates cytokine production^197,198^
	• Glutaminolysis; increased use of glutamine in absence of glucose^197,198^
	Regulatory T cell:
	• TCA; suppressive function^197^
	• Fatty acid oxidation; promotes Treg generation^197^
Dendritic cells	• Glycolysis; supports cell activation and proinflammatory phenotype (cytokines, chemokines) and rapid antibacterial response^199^
	• TCA/oxidative phosphorylation; supports phagocytic capacity, prolonged antihelminth response^199^
NK cells	• Glycolysis; supports killing, cell degranulation, Fas ligand expression and NKR-activated cell cytotoxicity. Important for IFNy production^200,201^
	• TCA/oxidative phosphorylation; needed for resting state cell survival and IFNy production upon activation^201^
Neutrophils	• Glycolysis; needed for neutrophil survival and functioning^202^
	• Pentose-Phosphate Pathway; needed for NET formation and production of superoxide^203^
B cells	• Glycolysis; needed for B-cell antigen receptor (BCR)-mediated growth/clonal expansion^204^
	• OXPHOS; restriction markedly impaired B cell growth and differentiation^204,205^

**Table 4:** Impact of metabolic pathways on adipocyte inflammation.

Cell type	Metabolic pathway and impact
Adipocyte	White adipose tissue:
	• Glycolysis; unlocks dormant adipocyte inflammatory vigor^19^
	• Fatty acid oxidation; supports reduction in triglyceride content and inflammation^206^
	Brown adipose tissue:
	• Glycolysis; needed to achieve maximum thermogenic output^207^

Various immune mediators can modulate inflammatory capacity, tissue healing, and viral pathogenesis, including type I IFN (e.g., IFNα, IFNβ) and type III IFNs (e.g., IFNλ).^134^ While both are necessary for regulation of inflammatory responses, type I IFNs are more often associated with worse disease immunopathology.^135,136^ Given the relevance of type I and type III IFNs in viral infections, it is not surprising that pathogen manipulation of these axes might play a role in disease pathogenesis.^137,138^ In fact, early activation of the Type I IFN axis through IFNAR/STAT signaling lead to decreased viral load and mild disease severity in SARS-CoV-2 infections^139^ and diminished viral replication and diversity in influenza infections in obese mice.^140^ In contrast, delayed activation of IFNs, but not other proinflammatory cytokines, resulted in poor disease progression during initial stages of SARS-CoV-2 infection.^141^ This initial delay results in elevated type I IFN responses in late stages of infection^142^ and increased and persistent viral load, inflammation, and severe disease outcome.^139,143,144^ Further, neutralizing anti-IFN auto-antibodies^145^ or inborn errors of type I IFN signaling^146,147^ were found in subjects that developed severe SARS-CoV-2-associated pneumonia. However, despite such advances, the role of type I IFNs in severe SARS-CoV-2 pneumonia is still controversial and requires further study,^148^ with most recent reports highlighting that SARS-CoV-2-induced type I IFN dysregulation is cell specific.^149^ Given that type I IFNs also impact adipocyte metabolism and inflammatory vigor,^19^ the ultimate effect these changes have on inflammation in obesity and viral infection should be investigated. As adipocytes are not “professional immune cells”, delayed and unchecked adipocyte inflammation might be a major contributor to the excessive inflammatory responses seen in obese individuals.

Few studies have examined the role of adipocyte-secreted factors in modulating immunosenescence leading to adverse infectious outcomes in obesity. Specifically, while obesity-associated chronic inflammation impairs cytokine signaling in hematopoietic stem cells, adiponectin treatment of obese mice restores proper stem cell proliferation and pathogen clearance.^150^ This suggests that adiponectin’s protective role may restrict basal inflammation via modulation of immune cell progenitor function. These protective effects could be diminished in obesity due to reduced adiponectin levels and negative correlation between adiponectin levels and immune cell sensitivity to LPS.^151^ The literature also supports the theory that the alteration of adiponectin secretion in obesity increases propensity for cytokine storm, with a focus on respiratory disease. Adiponectin promotes proliferation and wound repair of human bronchial epithelial cells^152^ and limits excessive lung inflammatory responses to invasive Aspergillosis in lean mice.^153^ Intuitively, an association between decreased adiponectin levels in obesity and immune cell hyperresponsiveness to influenza A has been suggested,^154^ highlighting the potential role of obesity-altered adipocytes.

Another key adipocyte-secreted mediator in disease pathogenesis is IL-6. The overproduction of IL-6 is an important risk factor for worse outcomes in H1N1 influenza,^155^ SARS,^156^ MERS,^157^ and SARS-CoV-2.^158,159^ Tocilizumab-dependent neutralization of IL-6 as a SARS-CoV-2 therapeutic is not fully elucidated. Randomized control trials of Tocilizumab therapy report both a reduction in likelihood of mechanical ventilation or death in patients with severe SARS-CoV-2 pneumonia^160,161^ and lack of a protective effect.^162^ These findings reveal that while IL-6 is linked with severe SARS-CoV-2-associated pneumonia, additional studies are needed to determine the best therapeutic approach to IL-6 inhibition, with potential requirement for cell-specific inhibition.^149^ Notably, adipocyte, but not AT macrophage, IL-6 secretion is the key regulator of WAT inflammation.^80^ Further, IL-6 secreted by adipocytes in response to inflammatory stimuli exacerbates responses to LPS-induced endotoxemia.^163^ Thus, adipocyte-derived IL-6 might play a role in the severity of SARS-CoV-2 and influenza-induced pneumonia seen in obese individuals.

Other infectious models also highlight the detrimental contribution of elevated leptin to inflammatory outcomes in obesity. Clinically, elevated plasma leptin levels are correlated with systemic inflammation in obese individuals,^164^ and leptin signaling through STAT3 promotes early inflammation associated with worse clinical disease during *C. difficile* infections.^165^ Experimentally, elevated basal leptin expression in obese mice correlates with increased parasite burdens in visceral leishmaniasis^166^ and exacerbated cytokine storm leading to increased mortality in response to *Francisella tularensis* infection.^104^ Elevated leptin levels contribute to lung tissue damage during H1N1 influenza infection via an IL-6 inducing mechanism.^167^ LCMV induces a pathologic memory T-cell response in the WAT that leads to adipocyte damage and reduced survival in obese mice. Intriguingly, the culpable memory T-cell population within the WAT is transcriptionally distinct from other tissues.^86^ Given that leptin limits proliferation and responsiveness of regulatory T cells while having opposite effects on effector T cells,^168^ inflammation-skewed adipocytes secreting excess amounts of leptin might play a role in the development of the culpable hyperinflammatory memory T cells, a phenotype that might extend to viral pneumonias in obesity.

## Adipocyte inflammation: future implications

Susceptibility to severe infection isn’t limited to the innate immune response to a pathogen. Obesity is also associated with decreased efficacy of vaccines. Inadequate levels of protective anti-Hepatitis B antibodies have been reported in obese individuals.^169^ Significant declines in protective levels of antibodies after immunization have been reported in obese individuals that received either the Hepatitis A or rabies vaccination.^170^ Vaccinated obese adults have twice the risk of influenza or influenza-like illness despite equal serological response to vaccination.^171^ Similar trends are seen in obese pediatric patients that receive tetanus vaccines.^172^ While these reports are limited in the breadth of vaccines affected by obesity, it is evident that the obese state negatively alters the adaptive immune system, and that bodyweight should be a considered variable during clinical trials of vaccines, most pertinently the SARS-CoV-2 vaccine. However, although direct mechanisms behind this observation remain unclear, contribution of AT inflammation in this setting should be considered. B cells that reside within AT are skewed toward a pro-inflammatory state during obesity, a state that limits their effector function and capabilities to produce antibodies.^173,174^ Obesity also decreases the numbers and diminishes activation of T follicular helper cells in peripheral lymphoid organs,^175^ potentially impacting proper development of mature B Cells. Finally, metabolism plays an important regulatory role in shaping the humoral immune response.^176^ Together, these observations invoke following questions: Does adipocyte inflammation play a role in B cell phenotypes within AT? Can adipocyte inflammatory capacity impact T and B cell metabolism and function in AT or in peripheral immune organs via secreted factors? If so, what impact does adipocyte inflammation have on vaccine efficacy, and importantly the efficacy of novel vaccines for pandemic strains of influenza and SARS-CoV-2?

The adipocyte is an important regulator of immune system homeostasis. During normal metabolic conditions, adipocytes modulate immune cells via their secreted mediators. The effect of obesity on adipocytes impacts the immune system’s capacity to respond to infection. On the other side of the spectrum, malnourished and underweight individuals are also more susceptible to infectious disease severity. While this is attributed to immune dysfunction due to immune cell nutrient starvation, recent advances suggest that decreased circulating levels of adipocyte-produced leptin play a significant role,^55^ and allude to adipocyte nutrient starvation impacting adipocyte-immune cell communication. Further, the suppression of murine adipocyte inflammation promotes metabolic disease.^177^ These findings suggest a basal level of adipocyte inflammation is critical to mount a proper immune response. Thus, being in a metabolic state that significantly alters adipocyte inflammatory capacity in either direction could be detrimental. Systematic reviews have revealed a “J-shaped curve”-distribution between BMI and risk of all-infection mortality^178^ and, notably, influenza-associated pneumonia,^179^ mirroring the relationship between obesity and all-cause mortality.^180,181^ While being slightly overweight (BMI 25-29.9) has been reported to be protective from infection-associated disease severity, further progression in increasing BMI (obese – BMI 30–34.9, morbidly obese – BMI 35 + ) increases susceptibility to severe infection.^180^ This is possibly attributed to obesity-associated inflammation having a detrimental effect on adipocyte function, either mitigating their protective capabilities and altering the immune response or driving protective traits (e.g., inflammatory response) to a point where they are harmful toward the host. If adipocytes are critical mediators of inflammatory responses, then maintaining a proper balance of AT inflammation is important, as being on either side of this “J-shaped curve” is unfavorable to host defenses against pathogens. Together, these observations invoke the following salient questions that address the immune contribution of adipocyte inflammation: Is there an ideal level of adipocyte inflammation that enhances immune responses to infection? How do we define the threshold where adipocyte inflammatory effects become detrimental to the host? Does activation of specific metabolic pathways in adipocytes regulate such threshold? Is adipocyte inflammatory capacity negatively impacting obese individuals combatting viral pneumonias such as influenza or SARS-CoV-2?

## Concluding remarks

**Fig. 2:**
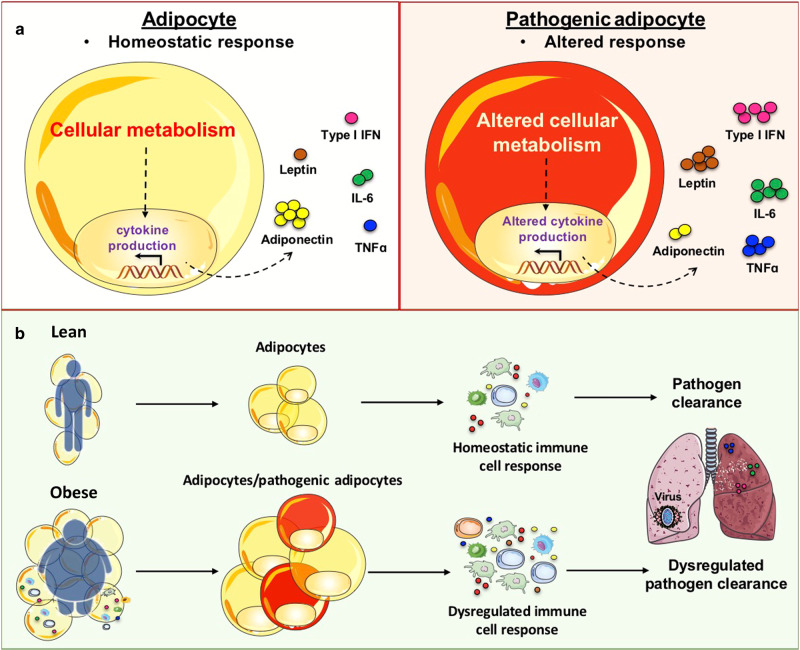
Pathogenic adipocytes contribute to impaired pathogen clearance in obesity. **a** Normal cellular metabolism regulates homeostatic cytokine production in healthy adipocytes. In contrast, altered cellular metabolism favors induction of a “pathogenic adipocyte” population that exhibit dysregulated cytokine and adipokine production which in turn amplifies disease severity. This pathogenic state could arise in obesity due to the altered metabolic and inflammatory microenvironment of adipose tissue. **b** Adipocytes contribute to homeostatic immune cell responses and pathogen clearance in the lean state. Obesity impacts adipocyte inflammatory phenotype, which could result in induction of pathogenic adipocytes. This results in dysregulated immune cell responses and culminates as altered pathogen clearance.

In summary, multiple pandemics (e.g., 2009 H1N1 Influenza, SARS-CoV-2) reveal obesity as an important risk factor for increased disease morbidity and mortality. Considered alongside the global trend toward a more obese population, it is imperative to develop a better understanding of the mechanisms behind obese susceptibility to infection. In this review, we highlighted a new and exciting area of long-neglected research: adipocyte metabolism and inflammation in viral pneumonias. Being skewed toward a proinflammatory state, alterations in adipocyte-intrinsic cellular metabolism impacts adipocyte-immune cell crosstalk via changes in adiponectin, leptin, and IL-6, as well as other pro-inflammatory mediators (e.g., TNF, IL-1, type I IFNs, etc.) is dysregulated during obesity. Published reports demonstrate the contributions of “pathogenic immune cells”^131–133,182^ and “sick fat”^17^ to inflammatory and metabolic disease. As not all obese individuals have the same susceptibility to metabolic disease due to the existence of healthy vs unhealthy WAT inflammation^183^ and obesity drives differentiation of pathogenic subsets of inflammatory Th17 cells,^184^ further studies examining adipocyte function in obesity and infection might reveal that a “pathogenic adipocyte” phenotype similarly contributes to disease severity (Fig. 2a). Importantly, as with immune cells, such adipocytes could have unique transcriptional signatures and functions that would serve as potential biomarkers for increased risk of infection severity in obesity. Through their inflammatory capabilities, “pathogenic adipocytes” may represent a critical underappreciated link between obesity, immune responses, and infection pathogenesis (Fig. 2b). Thus, defining the interconnected metabolic and inflammatory mechanisms that regulate adipocyte’s phenotype and contribution to obesity-associated immunomodulation and increased susceptibility to infection may provide a robust platform for discovery of novel biomarkers and therapeutic targets to reduce the clinical burden of infectious disease in an increasingly obese population worldwide.
